# A computational pipeline for functional gene discovery

**DOI:** 10.1038/s41598-021-03041-0

**Published:** 2021-12-07

**Authors:** Aolani Colon, Rishabh Hirday, Ami Patel, Amrita Poddar, Emma Tuberty-Vaughan, Tianyue Fu, Xin Ai, Wei Vivian Li, Li Cai

**Affiliations:** 1 Department of Biomedical Engineering, Rutgers University, 599 Taylor Road, Piscataway, NJ 08854 USA; 2 Department of Biostatistics and Epidemiology, Rutgers University, 683 Hoes Lane West, Piscataway, NJ 08854 USA

**Keywords:** Data mining, Data processing, Functional clustering, Gene ontology, Genome informatics, Literature mining, Computational biology and bioinformatics, Developmental biology

## Abstract

Many computational pipelines exist for the detection of differentially expressed genes. However, computational pipelines for functional gene detection rarely exist. We developed a new computational pipeline for functional gene identification from transcriptome profiling data. Key features of the pipeline include batch effect correction, clustering optimization by gap statistics, gene ontology analysis of clustered genes, and literature analysis for functional gene discovery. By leveraging this pipeline on RNA-seq datasets from two mouse retinal development studies, we identified 7 candidate genes involved in the formation of the photoreceptor outer segment. The expression of top three candidate genes (*Pde8b*, *Laptm4b*, and *Nr1h4*) in the outer segment of the developing mouse retina were experimentally validated by immunohistochemical analysis. This computational pipeline can accurately predict novel functional gene for a specific biological process, e.g., development of the outer segment and synapses of the photoreceptor cells in the mouse retina. This pipeline can also be useful to discover functional genes for other biological processes and in other organs and tissues.

## Introduction

**Figure 1:**
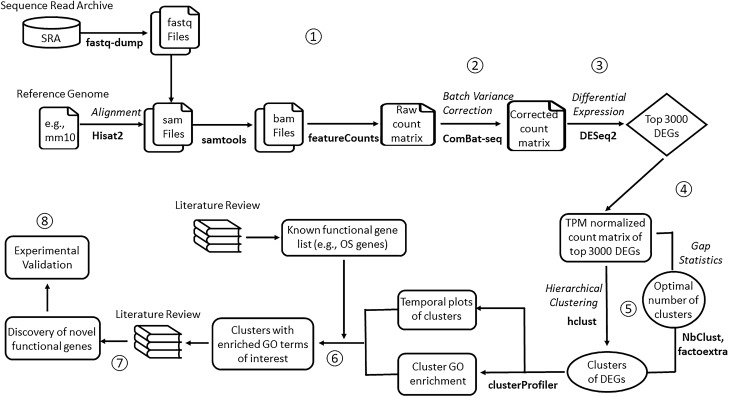
Outline of computational pipeline for functional gene discovery. Schematic diagram illustrates the steps in the computational pipeline for functional gene discovery in retinal outer segment development as an example: 1. Sequence alignment to generate raw count matrix, 2. Correction for batch effect/variance to generate corrected count matrix, 3. Detection of differentially expressed genes (DEGs), 4. Normalization, 5. Determination of the optimal number of gene clusters/expression patterns, 6. Gene ontology analysis, 7. Discovery of novel functional genes via literature search, and 8. Experimental verification of computationally predicted novel functional genes.

Many computational pipelines exist for the detection of differentially expressed genes (DEGs), but not for functional gene detection. Major steps for DEG identification include: (i) normalization, (ii) dispersion estimates, and (iii) differential gene expression based on condition. In order to carry out these functions, linux-based alignment tools, e.g., HISAT^1^ and STAR^2^, and R packages DESeq2^3^ and EdgeR^4^ are most commonly used. Few pipelines, however, move data along for further analysis beyond DEGs, such as gene ontology analysis and novel gene discovery^5^. We have developed a pipeline that covers the entire breadth of analysis for transcriptome profiling data, e.g., RNA-seq data, to facilitate functional gene discovery (Fig. 1). This pipeline takes raw data in the form of FASTQ file format and includes modules to align sequencing reads/tags from different datasets to a reference genome, adjusts batch effect, normalizes data based on condition, performs differential gene expression analysis, optimally clusters genes based on expression similarity, and carries out gene ontology enrichment analysis on each generated cluster. The identification of a new functional gene is based on (1) membership in gene cluster with established main biological function, e.g., photoreceptor outer segment (OS) development; and (2) the candidate gene does not have an established function with the main biological functions of the cluster. The functional gene discovery allows for the development of new hypotheses for the investigation of candidate genes. Key packages and methods utilized in this pipeline include Hisat2^1^, featureCounts^6^, ComBat-seq^7^, DESeq2^3^, the gap statistic method^8^, hierarchical/K-means clustering^1,3,9^, and clusterProfiler^10^. To validate the pipeline, we attempted to identify functional genes from publicly available datasets of mouse retinal development studies at NCBI GEO. We have compiled two datasets to generate a dataset of gene expression profiling from mouse embryonic day 11 (E11) to postnatal day 28 (P28), which cover the full length of retinal development. Our pipeline accurately and effectively identified new functional genes for the development of the photoreceptor OS.

## Results

### Sequence alignment, tag counting, batch variance correction and differential gene expression analysis

We tested our computational pipeline using two previously published RNA-seq datasets from mouse developing retinas (*Gene Expression Omnibus*, GEO accession # GSE74660^11^ and GSE101986^12^ under NCBI Short-Read Archive (SRA) # SRX1411331 to SRX1411346 and SRX3044650 to SRX3044673; see supplemental Table S1). We chose these two datasets for the following reasons: (1) they were generated using the same RNA extraction method; (2) samples were sequenced using the same platform GPL11002: Illumina Genome Analyzer IIx; (3) they were single-end read format; (4) the dataset GSE74660 contains samples from postnatal stages of the retinas, while the dataset GSE 101,986 contains both embryonic and postnatal samples; and (5) the sequence quality of all samples is high with > 93% tag mapping rate. All data samples were obtained via fastq-dump from the SRA toolkit (http://ncbi.github.io/sra-tools). The combined dataset contains 12 time points with at least two replicates for each time point. Each sample was then aligned to mouse genome (mm10) using HISAT2^1^, which resulted in > 97% seq tag mapping rate. The resulting SAM files were converted into sorted BAM files using samtools^13^. A count matrix of sequencing tags/reads with 33,487 genes (Supplemental Table S2) was generated using featureCounts^6^. To correct batch effect/variance from the datasets, we performed ComBat-seq analysis^7^, which resulted in an adjusted count matrix (Supplemental Figure S2 and Table S3). The adjusted count matrix was then analyzed by DESeq2^3^ to identify significantly differentially expressed genes (DEGs, p-value < 0.05) across the embryonic and postnatal development stage. Samples from E11 to E16 were considered as replicates for the embryonic stage, while samples from P0 to P28 were considered as replicates for the postnatal stage. The top ranked 3000 DEGs (Supplemental Table S4) were normalized using transcript per million (TPM) method^3^ and selected as the most significant genes in retinal development for further analysis.

### Clustering analysis and optimal cluster number selection by gap statistics

We next performed clustering analysis on the top ranked 3000 DEGs. A predominant clustering algorithm for temporal gene expression data is the hierarchical method^14^. In this method, the number of clusters is determined by the distinct number of branches on the dendrogram produced. However, this approach is highly subjective and inconsistent, hence we used the gap statistics method^8^ to determine the optimal number of clusters within the dataset. The gap statistics produced consistent results with varying input parameters and identified that the optimal number of gene clusters in this dataset was 30 (Supplemental Figure S2). K-means method was used in the gap statistics to determine the optimal K value. Thus, the top ranked 3000 DEGs were subsequently grouped into 30 distinct clusters with various members of similar expression pattern (Supplemental Figure S3 and Table S5).

### Identification of functional genes in clusters involved in photoreceptor outer segment development

For each of the 30 gene clusters, gene ontology analysis was performed using clusterProfiler^10^ to determine the enrichment of genes involved in a specific cellular component (CC), biological process (BP), and molecular function (MF). We further analyzed the clusters with gene expression level in rising trend, e.g., clusters #3 and 8 containing 512 and 124 genes, respectively. The expression pattern of the two clusters parallels with the development of the photoreceptor OS and synapses, which is known to begin around postnatal day 0 (P0) and peak between P10 and P14^11^.

**Figure 2:**
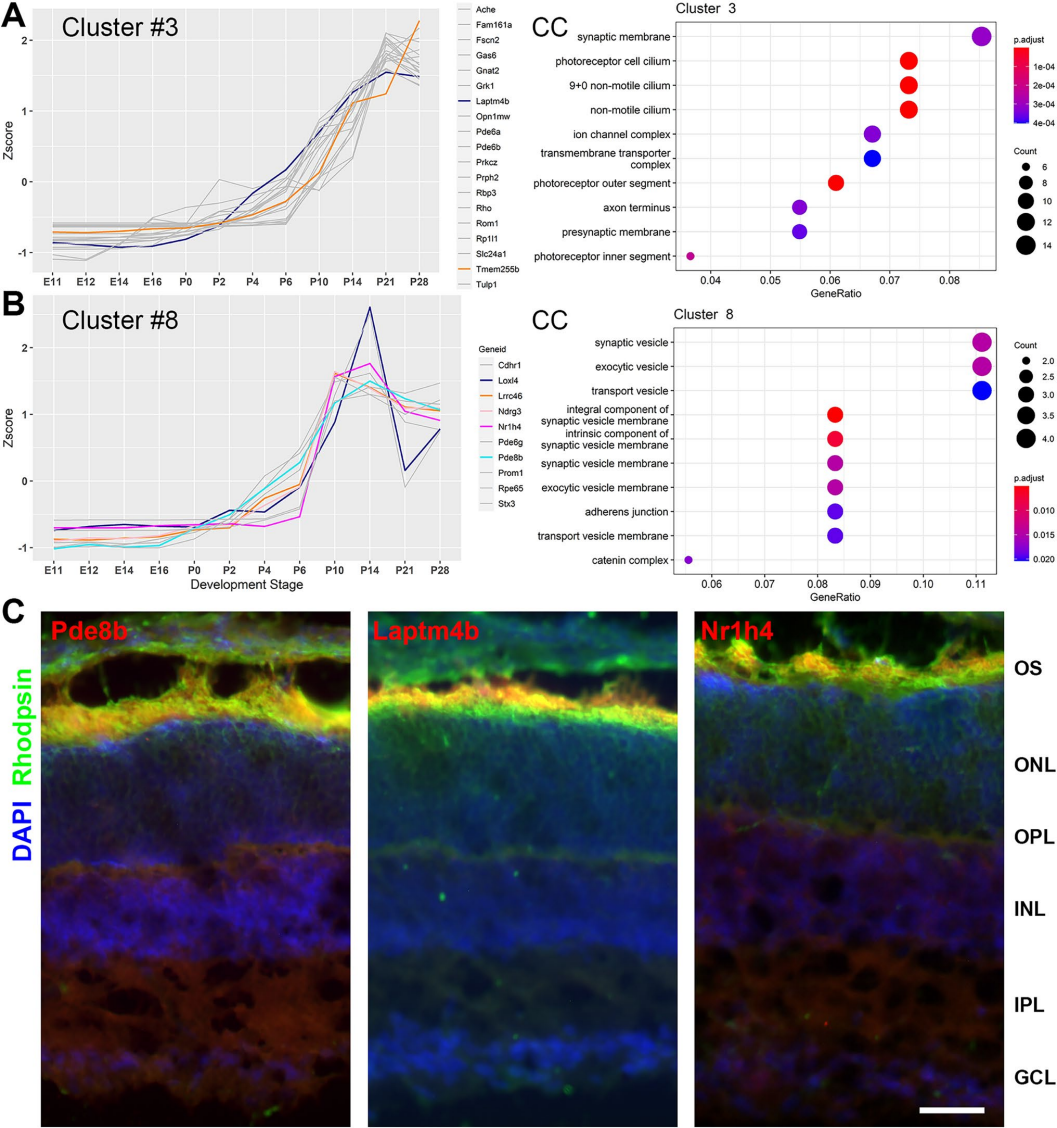
Gene expression profile and gene enrichment associated with OS development. Plots of gene expression level as Z-score over developmental stages and gene enrichment for clusters #3 (**A**) and #8 (**B**). Each line represents the expression profile of a gene. Gray lines represent known photoreceptor OS genes, while colored lines represent predicted novel functional OS genes. Gene ontology (GO) term enrichment for clusters #3 and #8 was plotted on the right (**A**,**B**). The size of the circle represents the number of gene counts, and color represents the significance ranked by p-adjusted value. GO terms (cellular component, CC) associated with the three clusters, ranked by p-adjusted value with top 10 processes were listed. (**C**) Representative photomicrographs of mouse retina sections at postnatal day 14 (P14) immunostained with antibodies against Pde8b, Laptm4b, Nr1h4, and known photoreceptor markers for rods (Rhodopsin). *OS* outer segment, *ONL* outer nuclear layer, *OPL* outer plexiform layer, *INL* inner nuclear layer, *IPL* inner plexiform layer, *GCL* ganglion cell layer. Scale bar 20 µm.

There were three clusters contain known OS genes (Supplemental Table S6) with GO term enrichment for photoreceptor OS development, i.e., clusters #2, 3, and 8. Thus, these three clusters were designated as OS gene clusters. Each gene from these clusters was then cross-referenced with a list of 126 manually curated known genes important for the development of the photoreceptor OS^15^ (Supplemental Table S6). This resulted in 25 DEGs (3 in cluster #2, 17 in cluster #3, and 5 in cluster #8) matched with the 126 known OS genes (Fig. 2A,B and Supplemental Table S7).

We speculate that other genes in these OS clusters could be involved in the OS development. Thus, each gene from these clusters was further analyzed for its function in the retina using PubMed literature searches. The genes that had no literature hits related to the photoreceptor or retina but with detected gene expression in the retina in VisiGene and GENSAT databases were then predicted as functional OS gene candidates. This resulted in two genes from cluster #3 (*Laptm4b and Tmem255b*) and five genes from cluster #8 (*Nr1h4, Loxl4, Lrrc46, Ndrg3, and Pde8b*), a total number of seven novel functional OS genes (see Distribution of predicted novel OS genes in clusters with K = 30 in Supplemental Table S8).

To determine whether the selection of optimal cluster number (K = 30) is significantly better than other choices of K, we tested K = 10 and 100 (Supplemental Figure S4). Although the known OS genes were also distributed in two clusters when using K = 10 as compared to K = 30, the total number of genes from the two clusters were significantly increased from 636 to 803 (see Comparison of cluster size with K = 10, 30, and 100 in Supplemental Table S7), making it more time consuming to perform literature search on more genes for functional gene discovery. When using K = 100, the known OS genes were distributed in 6 clusters, i.e., clusters # 3, 9, 17, 21, 23, and 62 with a reduced total number of genes (559) (see Distribution of known OS genes in clusters with k = 10, 30 and 100 in Supplemental Table S8). The decreased total number of genes could cause some of the novel functional genes be missing from these clusters.

### Experimental verification of computational identified functional genes

The top three candidate genes, *Pde8b*, *Laptm4b*, and *Nr1h4* from the two clusters were then experimentally validated to confirm their expression in the OS of the developing mouse retina. Immunohistochemical analysis was performed on tissue sections of mouse retina at P14 using antibodies against *Pde8b*, *Laptm4b*, and *Nr1h4.* Antibody against *Rhodopsin* was used as a positive control to stain the OS of rod photoreceptors. Developmental stage P14 was chosen for the two following reasons: (1) RNA-seq analysis reveals the expression level of these three genes was high at this stage, and (2) the mouse OS development reaches the peak level around P14^11^. Immunostaining results showed that signals from *Pde8b, Laptm4b and Nr1h4* antibody staining were detected in the OS and overlap with the signals from the known OS marker *Rhodopsin* (Fig. 2C). These results confirm the expression of the computationally predicted genes in the retina photoreceptors and validate the accuracy and effectiveness of the computational pipeline for novel functional gene discovery.

## Discussion

In this study, we have developed an effective and accurate computational pipeline that utilizes transcriptome profiling data for the identification of novel functional genes involved in a specific biological process, e.g., the photoreceptor OS development. This pipeline integrates bioinformatic tools and literature analysis on the existing knowledge for an accurate identification of novel functional genes. Our computational pipeline can also be applied to identify novel functional genes in other biological processes and in other tissue/organs. The expression of the candidate genes in tissue samples was further experimentally validated, demonstrates the effectiveness and accuracy of the pipeline. However, the explicit roles of these genes in the biologic systems, e.g., the development of the OS and synapses of the retina, are yet to be further experimentally investigated. Gain- and loss-of-function analysis, e.g., gene knockout or RNAi-mediated gene silencing experiments, should be conducted to explore the specific function of these genes. A limitation to this pipeline is that it requires manual curation of information on each individual gene within the cluster and it can be time consuming. In addition, datasets with significant batch effects or technical variations are not suitable to be combined and processed with our pipeline, as such datasets might not allow accurate detection of novel functional genes. In case there were a large number of genes lacking documented functions in a cluster, such cluster would not be able to provide a basis for novel functional gene prediction. Thus, it would be omitted from further analysis, and the downstream experimental verification (step 8) would not be feasible. Future direction of this pipeline should improve the manual curation process of gene information by leveraging automated text mining and machine learning technologies^16^ to expedite the literature review for novel functional gene discovery.

## Methods

### Development of the computational pipeline

The workflow of the computational pipeline is described in the following steps (Fig. 1):

1. Sequence alignment to generate raw count matrix
  For mapping next-generation sequencing data (e.g., FASTQ files) to a reference genome, we used a fast and sensitive alignment program, HISAT2^1^. Reference genome with the annotation file were obtained from the NCBI and UCSC Genome Browser. The resulting SAM files were converted into sorted BAM files using samtools^13^. Sequence tag counts were performed using featureCounts^6^ to generate a tag count matrix.
2. Correction for batch effect/variance
  This study contains two datasets (GSE74660 and GSE101986). To correct batch variance from the datasets, we performed ComBat-seq analysis^7^. ComBat-seq uses a negative binomial regression method to model batch effects and provide adjusted data by mapping the original data to an expected distribution^7^. It takes untransformed, raw count matrix as input for variance adjustment for RNA-seq samples from different batches. The adjusted data preserves the integer nature of counts and compatible with the current differential expression software (e.g., edgeR and DESeq2, which take untransformed raw count data as input). If datasets do not present a batch effect issue, e.g., datasets were from the same batch and in good quality, this step can be omitted in the pipeline.
3. Detection of DEGs
  DESeq2^3^ was used to identify DEGs in the transcriptome profiling data using default settings. The count matrix generated from the sequence alignment was used as the input for DESeq2. DESeq2 normalizes the counts of mapped reads and identifies DEGs by comparing an experimental condition to a control (e.g., embryonic time points for this study) to generate a list of the DEGs (Geneid) with associated values in baseMean, log2FoldChange, lfcSE, p-value, p-adj. In this study, we aim to identify functional genes for OS development, which is prominent during postnatal states. Thus, all postnatal time points were tested against embryonic time points to identify DEGs between these two developmental periods. The top 3000 DEGs identified through DESeq2 analysis were then further analyzed.
4. Normalization
  For clustering analysis, the dataset containing the top 3000 DEGs was normalized by converting the count matrix to a transcript-per-million (TPM) matrix using an algorithm in DESeq2^3^. For robustness, the median TPM was used across all replicates to represent gene expression level at that time point. The TPM values were then transformed into z scores by subtracting the mean of the genes and dividing by the standard deviation.
5. Clustering analysis with optimal number of gene clusters
  Hierarchical clustering was performed on the top normalized genes. We chose to use hierarchical clustering since it is both fast and interpretable. Due to its hierarchical structure, clusters can be easily merged or divided based on gene expression patterns with a cluster number different from the one identified by the gap statistic. Soft clustering allows the calculation of probability for genes with ambiguous assignment. However, to perform downstream enrichment analysis, a threshold is still needed to convert the probabilities to binary results. If ambiguous genes are of interest, users can re-run the clustering step of the pipeline with different cluster numbers to gain a consensus about the assignment of these genes. The clusters were created using the Euclidean distance and the complete linkage method. These parameters were chosen as they resulted in more well-balanced clusters compared to the other methods. Temporal plots were further utilized to visualize the expression of the genes in each cluster.
  To identify the optimal number of clusters or gene expression patterns within the dataset, we used the gap statistic method^8^. The gap statistic compares the within-cluster dispersion with that expected under a reference null distribution^8^. Therefore, a larger gap statistic indicates a better cluster number for a given dataset. After testing the data in a varying number of clusters up to 100 possible clusters (kmax = 100), the optimal cluster number (K = 30) was selected using the gap statistic plot (Supplemental Figure S2).
6. Gene ontology analysis of genes in the clusters
  Genes in each cluster were further analyzed based on adjusted p-values with default configurations to determine the enrichment on the biological process (BP), cellular component (CC), and molecular function (MF) via clusterProfiler^10^. The enrichment of gene ontology (GO) terms of specific biological function, e.g., development of the OS, cilium and synapse (clusters #3 and #8) in each cluster were then selected for novel functional gene discovery.
7. Discovery of novel functional genes
  To predict a novel functional gene, we performed literature search for each gene in those clusters involved in a particular term in the BP, CC, and MF of interest. The predication of a novel functional gene is based on 1) its membership in a cluster with established biological function, e.g., development of photoreceptor OS and synapses, and 2) lack of documented function from literature search. For example, Pde8b is a member in cluster #2, while literature search using PubMed resulted in zero hits with the terms: “Pde8b” and “retina” or “photoreceptor”.
8. Experimental verification of computationally predicted new functional genes
  Techniques including immunohistochemistry, qRT-PCR, and in situ hybridization are commonly used for the detection of gene expression. In this report, CD1 mouse retinas at P14 (n = 3) were harvested and processed following a previously established protocol^17^. Briefly, retinas were dissected from the animals and fixed with 4% (w/v) PFA for 1 h. Following fixation, samples were washed three times with PBS for 10 min each, soaked in 15% sucrose overnight and then 30% (w/v) sucrose at 4 °C until the retinas sank, embedded in cryopreserving media (Tissue Tek OCT compound), and stored at − 20 °C. Cryosectioning was performed with a CryotomE (Thermo Scientific, Waltham, MA). Sample Sects. (12 mμ thickness) were air dried for 10 min and stored at − 20 °C. Immunofluorescence staining was performed on sections as described previously^17^ using antibodies against *Pde8b*, *Laptm4b, Nr1h4*, and *Rhodopsin*. Cell nuclei were counter stained with DAPI to highlight the retinal structure. Images of the immunostained sections were captured using a Zeiss Axio Imager M1 (AxioVision 4.8 software) and a Zeiss LSM 900 (Zeiss ZEN blue software).

## Supplementary Information

Supplementary Information 1.

Supplementary Information 2.

Supplementary Information 3.

Supplementary Information 4.

Supplementary Information 5.

Supplementary Information 6.

Supplementary Information 7.

Supplementary Information 8.

Supplementary Information 9.

Supplementary Information 10.
