# Supplementary material for: A computational pipeline for functional gene discovery
Source: Sci Rep. 2021 Dec 7;11:23522. doi: 10.1038/s41598-021-03041-0 (PMC8651667; doi:10.1038/s41598-021-03041-0)
Supplement: Supplementary file 2 — Supplementary Information 2. [file 41598_2021_3041_MOESM2_ESM.pdf]

SUPPLEMENTARY INFORMATION

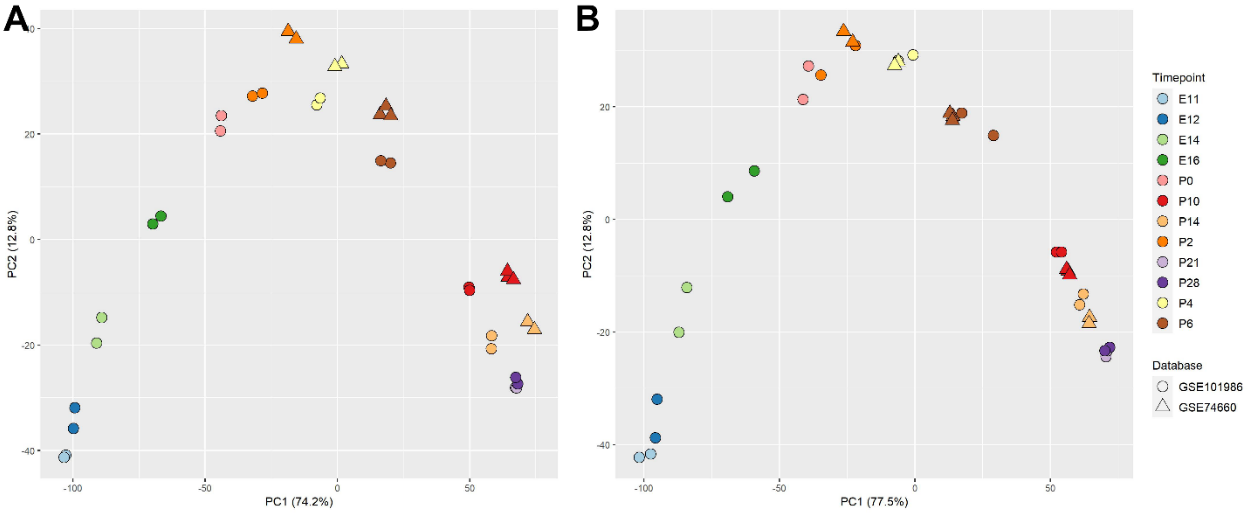

Figure S1. PCA plots of RNA-seq samples from two different datasets.
PCA plots of RNA-seq samples from two different datasets with GEO accession # GSE101986 and GSE74660 before (**A**) and after (**B**) batch effect correction.

A

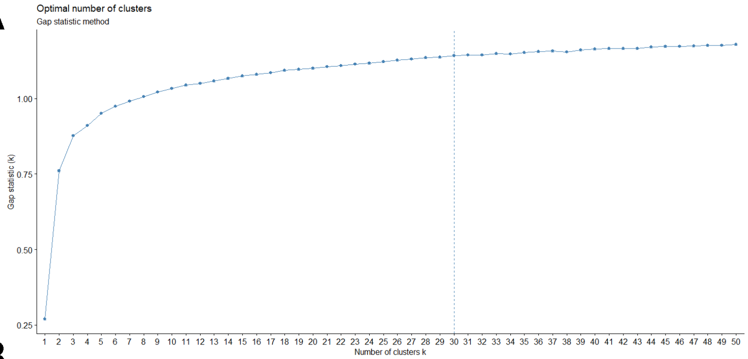

B

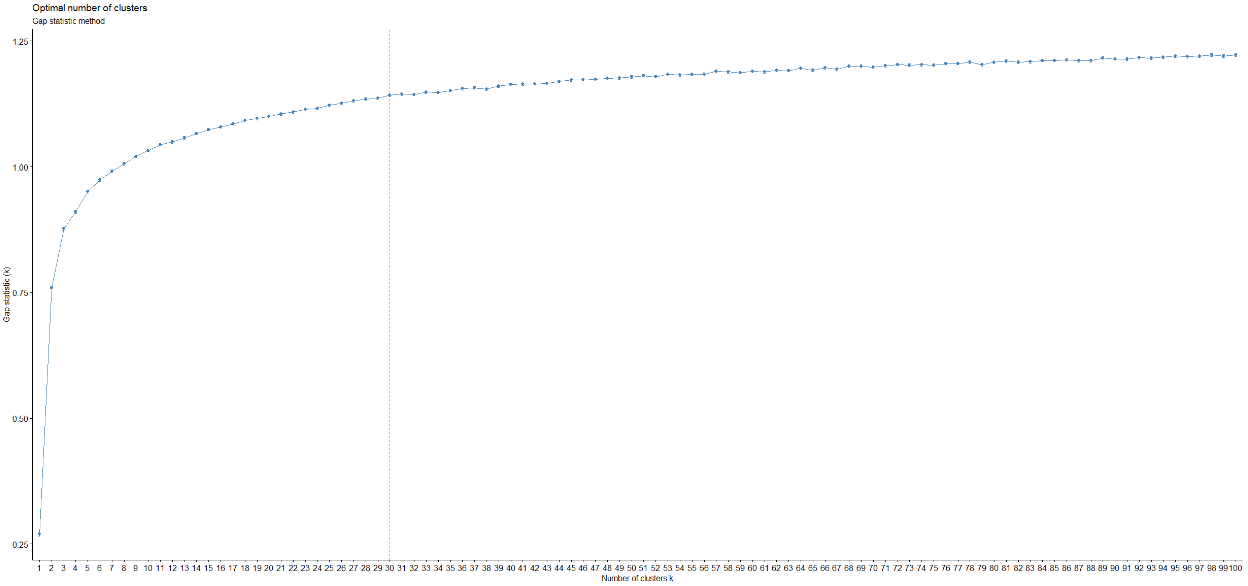

Figure S2. Plots of the optimal number of clusters (Kmax=50 and 100) as determined by gap statistic method. The optimal cluster size of 30 was chosen based on the gap statistic cutoff, where the difference of gap statistic value between two clusters is at a minimum. The x-axis is the size of clusters (number of clusters) and y-axis is the gap statistic value. Plots of gap statistics with kmax = 50 (**A**) and 100 (**B**).

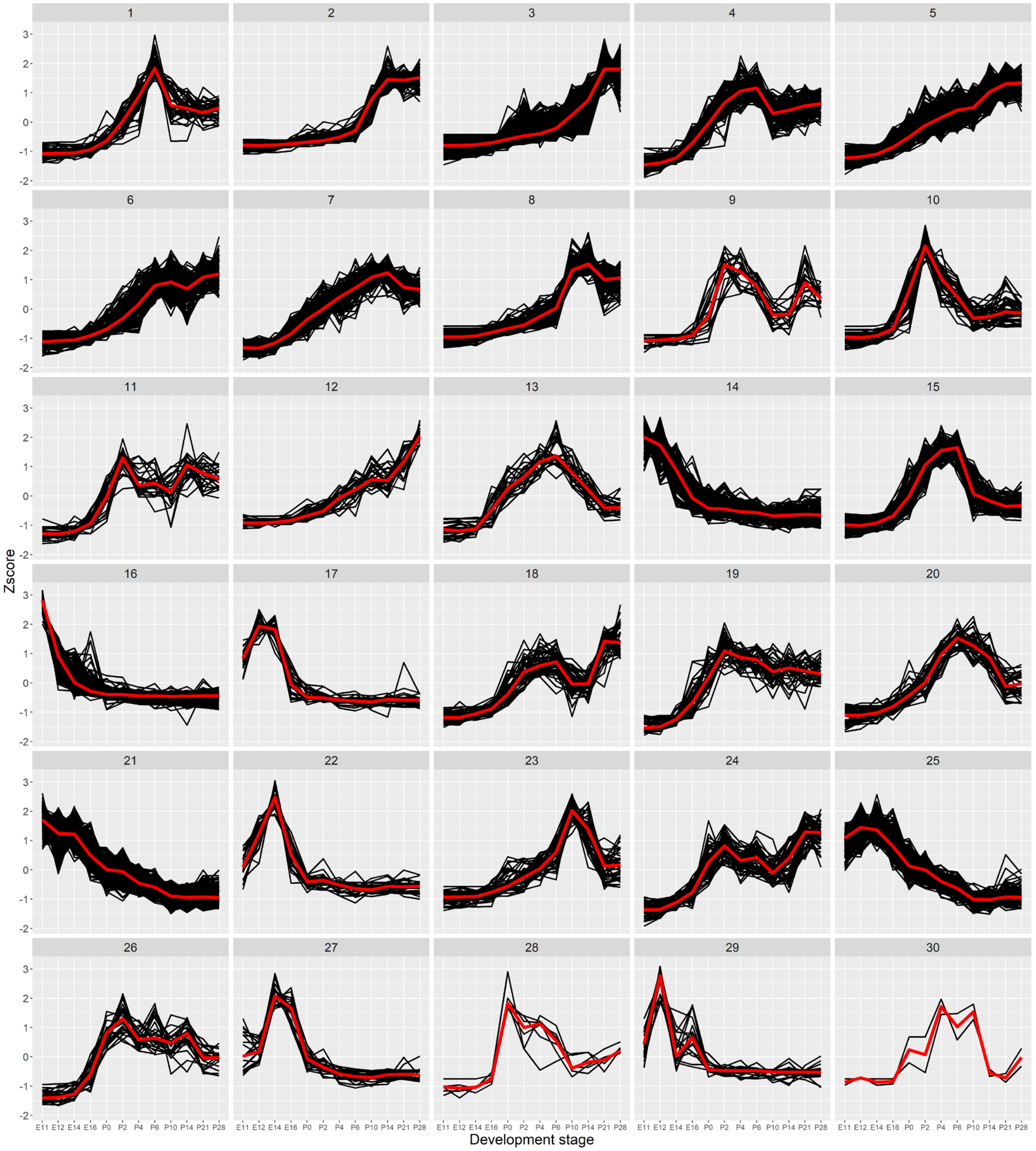

Figure S3. **Plots of 30 clusters from the top 3000 normalized DEGs**. The x-axis represents the developmental stages from embryonic day 11 (E11) to postnatal day 28 (P28). The y-axis represents the z-score, a normalized level of gene expression.

A

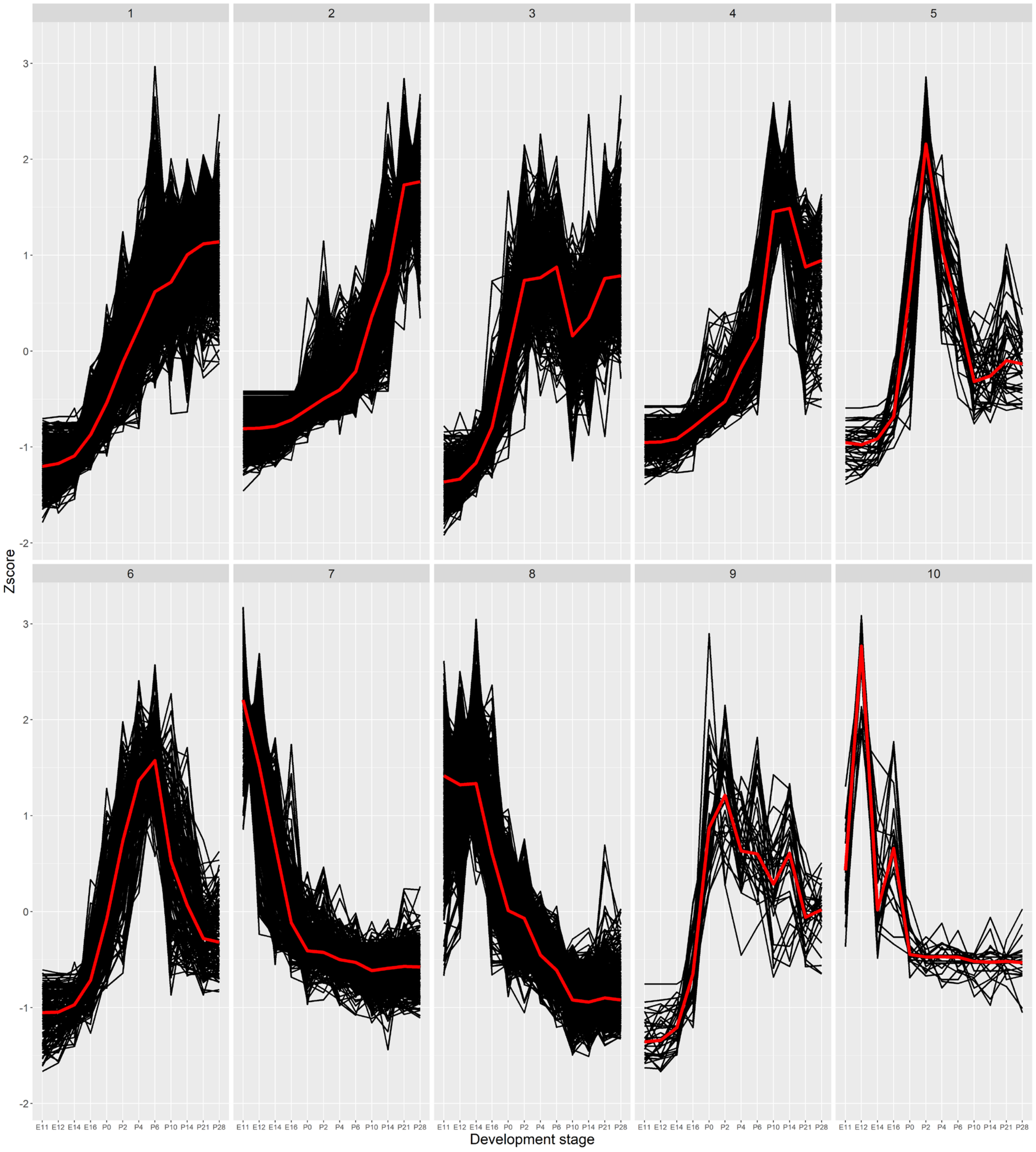
1
2
3
4
5
6
7
8
9
10
Zscore
3
2
1
0
-1
-2
E11 E12 E14 E16 P0 P2 P4 P6 P10 P14 P21 P28
Development stage

B

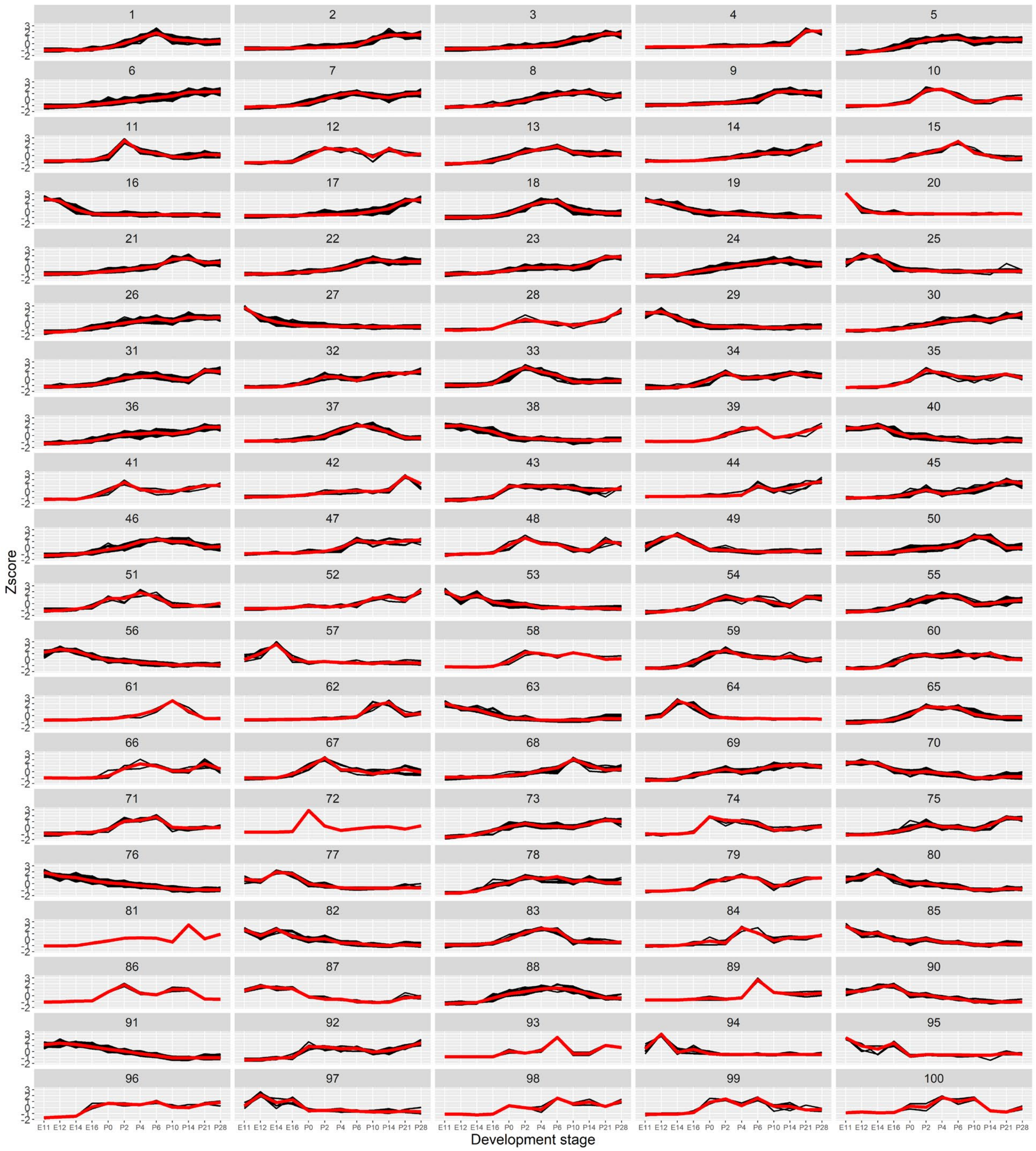

Figure S4. **Temporal plots of the top ranked 3000 DEGs**.
Plots with K = 10 (**A**) and 100 (**B**). The x-axis represents the developmental stages from embryonic day 11 (E11) to postnatal day 28 (P28). The y-axis represents the z-score, a normalized level of gene expression.

Supplementary Tables

All supplemental tables are uploaded to the journal website as attachments to this manuscript:

Table S:. List of RNA-seq samples from two batches included in this study

Table S2: Raw count matrix from combined RNA-seq datasets

Table S3: Corrected count matrix from combined RNA-seq datasets

Table S4: List of top ranked 3000 differentially expressed genes

Table S5. List of membership of the top 3000 DEGs in 30 clusters.

Table S6. List of genes known to be important for retinal outer segment development.

Table S7. Clusters #3 and 8 contain the highest number of known OS genes and related GO terms.

Table S8. Distribution of known and predicted OS genes in each different clusters with K = 10, 30, and 100.

Supplementary Source Code

Codes (R scripts) used in this study are available for download in a file named “scripts.zip”.
